# Soft Ionic Pressure Sensor with Aloe Vera Gel for Low-Pressure Applications

**DOI:** 10.3390/mi13020146

**Published:** 2022-01-18

**Authors:** Vishnu Sujeesh, Godwin Ponraj, Hongliang Ren

**Affiliations:** 1 Department of Biomedical Engineering, National University of Singapore, Singapore 117575, Singapore; vishnusujeesh@u.nus.edu (V.S.); godwin.joseph@u.nus.edu (G.P.); 2 Department of Electronic Engineering, The Chinese University of Hong Kong, Hong Kong

**Keywords:** pressure sensing, aloe vera gel, soft sensors

## Abstract

Ionic pressure sensors are made of ionic compounds suspended in a suitable solvent mixture. When external pressure is exerted on them, it is reflected as a change in electrical parameters due to physical deformation and a redistribution of ions within the sensing medium. Variations in the composition and material of the sensing medium result in different pressure sensors with varying operating ranges and sensitivity. This work presents the design and fabrication procedure of a novel soft-pressure sensor for a very low-pressure range (<20 mm Hg) using Aloe vera gel and Glycerin as the solvent for the ionic sensing medium. We also provide a comparative study on the performance of sensor prototypes with varying solvent concentrations and geometric parameters based on a series of characterization experiments. Maximum sensitivity ($7.498\times 10^{-4}\;\Omega /\mathrm{mmHg}$) was observed when using 40% glycerin in the sensing medium, filled in a toroidal geometry with outer and inner channel diameters of 8 mm and 7 mm, respectively. The proposed sensor is entirely soft and can be designed to conform to any desired geometry.

## 1. Introduction

Pressure-sensing has various applications in several domains, from biomedical engineering to industrial automation. The requirement of each application is often uniquely characterized by the requirement of the resolution, sensing range and sensitivity from the sensor. Such characteristic parameters of a pressure sensor are influenced by several factors, including its working principle, sensing material properties, geometry, etc. [1,2]. Based on the materials used in their fabrication, they can also be classified as rigid or soft sensors. While the hard-encased rigid pressure sensors are suitable for industrial and conventional robotic applications, soft pressure sensors, made up of entirely soft materials, find their application in biomedical devices, wearable sensors, and soft robotics [3]. Soft sensors have also been better sensitive in low-pressure ranges than their rigid counterparts [1] due to their ability to conform and engage more with the forces and pressures in their vicinity. An application example for the low-pressure range sensing is the measurement of Intracranial pressure (ICP), where the normal range is usually around 10–15 mmHg for healthy adults, 3–7 mmHg for children, and 1.5–6 mmHg for neonates [4]. ICP is a clinically measured parameter to monitor neurological and neurosurgical conditions in patients [5]. Higher ICP could indicate various conditions such as lesions, tumors, cerebrospinal fluid (CSF) circulation problems, etc. [6].

Sensors for a low-pressure range can be realized by designing specialized microstructures or using elements and materials that are inherently responsive to a low-pressure stimulus or a combination of both. Even though microstructures have been proven to have higher performance [7], they often require specialized fabrication processes such as MEMS micromachining. While using specialized materials does not generally require complicated fabrication, their sensing performance depends on the material properties. Hence, studies in this area have focused on altering the composition of the chief sensing material to achieve the optimal resolution and sensitivity in the desired pressure range.

In this work, we propose the use of a novel combination of aloe vera gel, glycerin, and salt (NaCl), to develop a soft pressure sensor for very low range (<20 mmHg or <2.67 kPa) applications. Aloe vera gel is a highly viscous liquid obtained from a fleshy succulent plant known as Aloe vera (syn. *Aloe barbadensis* Mill., Fam. Liliaceae). It has been used worldwide for centuries due to its medicinal value. The major component of aloe vera gel is water (99.1%), while plant cells form the remainder of its composition [8]. The selection of Aloe vera gel as a base material for the sensing medium is motivated by two reasons. Firstly, it is a naturally available eco-friendly resource that can provide a greener alternative to the existing synthetic chemical-based ionic gels. Secondly, Aloe vera leaves are known for their electrical anisotropy—i.e., their electrical conductivity is not uniform in all directions. Previous research has shown that application of higher voltage gradient has registered significantly reduced resistance values in directions along the length of the leaves than across the width of the leaves, suggesting ion channels in conducting bundles [9]. Similarly, it has also been shown that increased aloe gel concentration and higher ohmic heating (indirectly related to voltage gradient) results in higher electric conductivity of the gel [10]. Both these results suggest that aloe vera gel inherently contains electrolytic ions (such as Ca, K, Mg, and Na [11]) that can be leveraged into developing a more sensitive pressure sensor without adding a large number of external ions.

In contrast, most existing ionic gel sensors have a neutral medium with conductive ions added separately via complex polymerization procedures [12,13]. Hence, only a small quantity of NaCl was added to further enhance the ionic concentration of the aloe gel medium. NaCl dissolves in the gel and provides additional anions (Cl^−^) and cations (Na^+^) for the ionic redistribution in the medium. When external pressure is applied, the geometric change and the redistribution of ions in the medium change the overall resistance of the sensor. Glycerin is used as a thinning agent to modify the viscosity and increase the workability of the aloe vera gel.

For low-pressure range applications, a sensor’s sensitivity can be regarded as the most important parameter used to evaluate its performance. Hence, variations in the composition of the sensing material, geometrical size and shape of the sensor were analyzed to identify the optimum solution to achieve high sensitivity. A prototype of the proposed sensor was also compared with an AgNW-based sensor to understand the piecewise linear proportional relationship between the applied pressure and sensor output over a wider range. The proposed aloe gel-based sensors showed good linearity and sensitivity at less than 20 mmHg pressure inputs.

The rest of the manuscript is organized as follows. Section 2 contains a brief account of existing soft pressure sensors, base materials and working range. Section 3 explains our proposed sensor design, fabrication and introduces the design parameters considered for the optimization problem. Section 4 describes the characterization experiments performed on the various prototypes and compares their results to identify the optimal solution. A summary of the work performed, along with some recommendations for future improvement, are provided in Section 5.

## 2. Related Work

In general, pressure sensors are transducers that convert a mechanical input (pressure) into an electrical output (capacitance, resistance, etc.). Thus, apart from being classified as rigid and soft sensors, they can also be classified based on their working principle as capacitive, resistive, piezoelectric, optical, ionic fluid-based, etc. This section uses a few representatives from corresponding pressure sensor types and discusses their fundamental working principles.

Capacitive sensors work by changing their capacitance in response to changes in pressure. The capacitance change occurs when a dielectric layer sandwiched between two conductive layers is compressed from the pressure. Microstructures can increase the sensitivity of the sensors, as shown in the carbon nanotube coated elastomer fibre-based capacitive pressure sensors in [7]. Furthermore, the size and frequency of specific microstructures can be tailored for custom pressure-sensing requirements. An example can be seen in [14], where micro-structured pyramids were cast out of polydimethylsiloxane (PDMS) and deposited with Au to form conductive layers of a capacitive pressure sensor. Both these sensors are highly sensitive in low-pressure ranges of under 20 kPa. However, capacitive sensors are more susceptible to external noise and require dedicated capacitance readout electronics. As such, capacitive sensors are preferred in applications that require high sensitivity and can afford the power and cost for operating the accommodating electronics.

In resistive pressure sensors, the external pressure changes cause the active conducting material to change its resistance in response. Thus, they behave similarly to a variable resistor and easily measure simple voltage divider or bridge configurations. An ultrathin, flexible, sterilizable, and nonferromagnetic low-pressure range sensor was presented in [15]. The sensor consists of carbon nanotube (CNT)-filled Polyvinylidene fluoride (PVDF) as the main sensing material, with sputtered copper electrodes and a Kapton polyimide substrate. Another work presented a piezoresistive pressure sensor using Liquid Crystal Polymer (LCP), a thermoplastic polymer that contains links of rigid and flexible monomers [16]. It uses a Wheatstone bridge in a half-bridge configuration to measure resistance changes when external pressure is applied. Other materials used to fabricate piezoresistive pressure sensors include Poly(styreneethylene-butylenestyrene) (SEBS) [17], Polyacrylamide hydrogel [18], PDMS, Ecoflex, etc., as substrate or base materials and graphene nanofibers [19], porous carbon flowers [17], AgNW, zinc powder, etc., as conductive and sensing materials. Of these, AgNW-based sensors are known to have good sensitivity in the lower pressure ranges (0.8 to 2.1 kPa) [20], (<6 kPa) [21]. Hence, they are used to compare the proposed aloe vera-based sensors’ sensitivity and linearity within (<2.6 kPa) and beyond their intended range.

Resistive pressure sensors can also be made using an ionic-liquid-based sensing medium. The resistance change will be due to the change in the redistribution of the ions in a liquid medium. There are several examples in the literature of such sensors, with the main difference being the choice of conductive fluid. Most of them use NaCl solution, with additives such as glycerol [22,23] or ethylene glycol [24] to increase the viscosity and performance of the ionic fluid. Some ionic sensors can also be characterized so as to be interpreted from multiple parametric variations. For example, an ionic skin that can measure compressive strain from different output signals such as open-circuit voltage and short circuit current, in addition to the conventional resistance and capacitance values is reported in [25].

Piezoelectric pressure sensors are active sensors. They differ from resistive and capacitive sensors because they generate electrical current or voltage in response to external stimuli. Specific piezoelectric materials such as Lead Zirconium Titanate (PZT), form the sensing medium in such sensors [26]. They can generate open-circuit voltages on the order of tens of mV in response to pressure changes. Recent studies have also presented novel concepts for the development of soft pressure sensors, e.g., using perovskite quantum dots in fibre-optic fabry-perot sensors [27], using hydrophobic microfluidic channels in combination with ultrasonic imaging systems [28], etc.

We propose a novel ionic-chief sensing medium consisting of Aloe vera gel, NaCl, and Glycerin. Our proposed sensor in this work can be categorized under the ionic-gel-based resistive pressure sensor. They can be easily fabricated using a 3D printed mold without involving complex steps such as photolithography or other MEMS procedure. Furthermore, it does not require specialized electrical readout circuitry for its operation.

## 3. Materials and Methods

**Figure 1:**
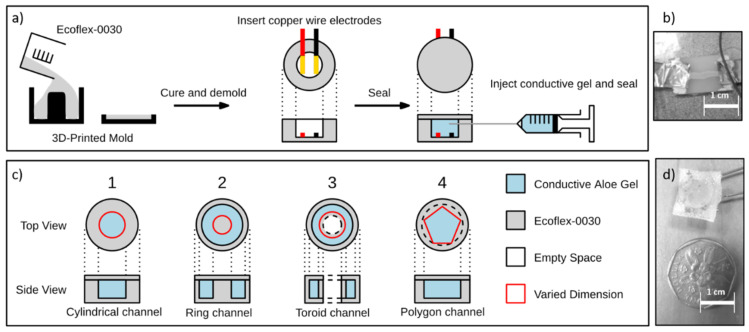
(**a**) Fabrication procedure of the proposed aloe vera gel based pressure sensor. (**b**) Prototype of AgNW sensor used in initial comparison experiment with the proposed aloe vera gel sensor. (**c**) Parametric variations considered related to the geometry of the sensing medium channel. (**d**) Prototype of aloe vera sensor kept near a Singaporean one dollar coin for size comparison.

The proposed aloe gel sensor consists of two main components, the sensing gel and the elastomer body that deforms in response to pressure acting on it. The sensing gel was fabricated by mixing aloe vera gel and salt in a 10:1 ratio by weight. As mentioned in Section 1, since aloe vera gel inherently has electrolytic ions, the ratio of external ions (salt) added to the medium is less than for other existing ionic gel sensors (Ex: solvent-ionic compound ratio by weight in [12] is 18:3). A drop of blue food coloring was added to enhance the visibility of the sensing gel once encased in the elastomer. Ecoflex 00-30 (Smooth-On Inc., Macungie, PA, USA), a two-part platinum cured elastomer, was used in making the elastomer body. The two-part elastomer components were mixed and cured in a 3D printed mold to form a soft cylindrical container with 2 cm outer diameter and 1 cm height. Before the sensing gel was poured in, wires were inserted to form an electrical connection. The filled elastomer container was sealed with a thin layer (2 mm approx.) of ecoflex to form the aloe gel sensor. Figure 1a shows the overall fabrication procedure of the proposed aloe gel sensors.

When external pressure is applied to the sensor, the sensing medium undergoes physical deformation due to the compressive force. Since the resistance of a conductor depends on its dimensions, the change in geometric parameters of the medium under pressure indirectly changes its resistance. Furthermore, the application of pressure could result in the redistribution of ions within the sensing medium, which also indirectly changes the resistance of the medium. The applied pressure is thus represented as a change in resistance of the sensor. This change can be measured by a multimeter for standalone sensing or by realizing a voltage divider or Wheatstone bridge setup for incorporation into the control systems.

AgNW (Silver Nanowire)-based pressure sensors were fabricated to compare the linearity of the proposed aloe gel sensors. They comprise a thin film of AgNW, drop cast on an elastomeric substrate’s top surface. Ecoflex-0050 was mixed and cured in a thin sheet with a thickness of approximately 2 mm in a Petri dish. The cured layer was cut into a rectangle of 2 cm by 1 cm. The surface of the ecoflex was cleaned with isopropyl alcohol, and an AgNW suspension containing silver nanowires was suspended in water with a cellulose binder dropped cast using a dropper in a thin layer lengthwise across the elastomer. After the AgNW layer dried, silver paste was applied to both the ends of the sensor and conductive copper tape electrodes were stuck to the paste. The silver paste cured and firmly attached the electrodes to the sensor. Finally, another layer of ecoflex of equal thickness was cast onto the sensor and allowed to cure again. Figure 1b shows the prototype AgNW sensor fabricated using the above-mentioned procedure.

A standard size of 1 cm diameter and 0.7 cm thickness was used for the optimization experiments for all the prototypes. Five parametric variations and four values were considered for each parameter, resulting in a total of 20 sensor prototypes being fabricated. The first parameter is the concentration of the glycerin used in the sensing medium. Four prototypes were made, each with different concentrations of glycerin mixed into the aloe gel by weight (20%, 40%, 60%, 80%). The remaining four parameters are related to the geometry of the well or channel containing the sensing medium. They are the diameter of the central cylindrical channel, the size of the ring-shaped channel, the toroid-shaped channel (with center removed), and the polygonal shape of the central channel. The cylindrical channel prototypes have a central cavity in their elastomer body in the shape of a cylinder with a varying diameter (1 mm increments from 5 mm to 8 mm) (Figure 1(c1)). The ring and toroidal prototypes have a ring-shaped channel in their elastomer body with varying inner diameters (1 mm increments from 4 mm to 7 mm). The difference between them lies in the central region of the sensor. While the ring sensor centers are filled with the elastomer body, the toroidal geometry had hollow cavities at the center (Figure 1(c2,3)). The final parametric variation produced four different shapes (hexagon, pentagon, square and triangle) for the well to contain the sensing medium (Figure 1(c4)).

The prototypes were made by casting different molds that generated different geometries for the sensing channel. Once cast, each sensor was demolded, 30 AWG copper wire was inserted before the sensing gel was poured and the sensor was sealed with ecoflex. The sensors were left undisturbed for 24 h to cure fully before testing. Figure 1c shows a summary of the different geometries fabricated and Figure 1d shows the size comparison of one of the prototype sensors with a $1 coin (Singaporean Dollar).

## 4. Results and Discussion

### 4.1. Comparison between AgNW and Aloe Sensor

The initial comparison between the AgNW and the proposed sensor was made beyond the intended range (0 mmHg to 50 mmHg) to better understand the sensitivity and linearity of the sensors.

**Figure 2:**
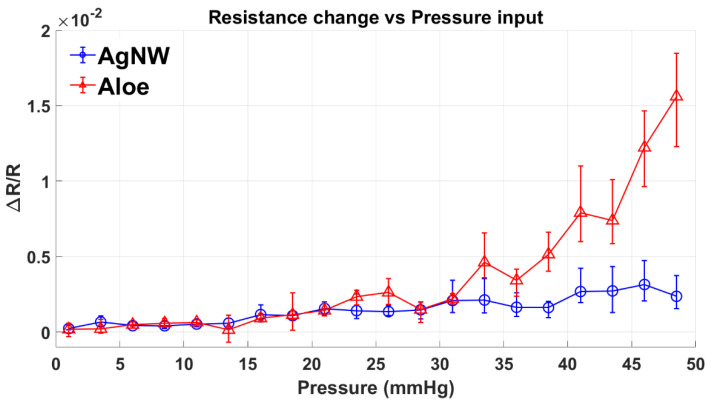
Comparison of response between AgNW and Aloe sensors. Aloe sensor exhibits piecewise linearity with sensitivity values of 5.46 × 10^−5^ mmHg^−1^, in 0–20 mmHg range and 44.49 × 10^−5^ mmHg^−1^, in 20–50 mmHg range, while the AgNW sensor is linear in the entire 0–50 mmHg range with a sensitivity of 5.41 × 10^−5^ mmHg^−1^.

The aloe vera-gel sensor exhibited piecewise linearity with higher sensitivity in the 20–50 mmHg range (44.489 × 10^−5^ mmHg^−1^) over the 0–20 mmHg range (5.4567 × 10^−5^ mmHg^−1^). However, repeated sensor testing in the higher pressure range deteriorated its performance. One reason for this could be the rapid degradation of the aloe-based gel, which has very low viscosity once it encounters the salt. The AgNW sensor showed a more linear pressure sensing behaviour than the aloe-gel sensor, with a sensitivity of (5.4052 × 10^−5^ mmHg^−1^). Comparing the performance of both the sensors, the aloe-gel sensor exhibited better sensitivity than the AgNW sensors in the higher pressure range (Figure 2). However, due to the deterioration problem, subjecting the sensor to repeated loading of a pressure higher than 20 mmHg (2.6 kPa) was not practical. In the lower range, the performance of both sensors was identical. Thus, in the following optimization experiments, the applied pressure was limited to a range of 0–20 mmHg.

### 4.2. Characterization Experiments for Parameter Optimization

The pressure input for the sensors was applied using an INSTRON Universal Testing Machine (UTM) (Illinois Tool Works Inc., Chicago, IL, USA). The resistance change of the sensors was measured using a digital multimeter. In addition to the characterization experiments to find the sensitivity of the sensors, three other experiments were conducted. For the experiments that followed, the Instron UTM was programmed to apply specific compression force for a specific time interval, such that the sensors are subjected to the pressure profile defined by the experiments. The step response of the sensor was recorded by applying pressure in incremental steps of 5 mmHg. This experiment analyzes the sensor response for the same input pressure during the loading and unloading. The next experiment subjected the sensors to repeated cycles of full load (20 mmHg) and no-load conditions alternatively. Finally, the sensors were subjected to a constant load of 20 mmHg for a prolonged duration of 30 min to observe any drift in the output parameter.

#### 4.2.1. Varying Viscosity and Composition of Sensing Gel

**Figure 3:**
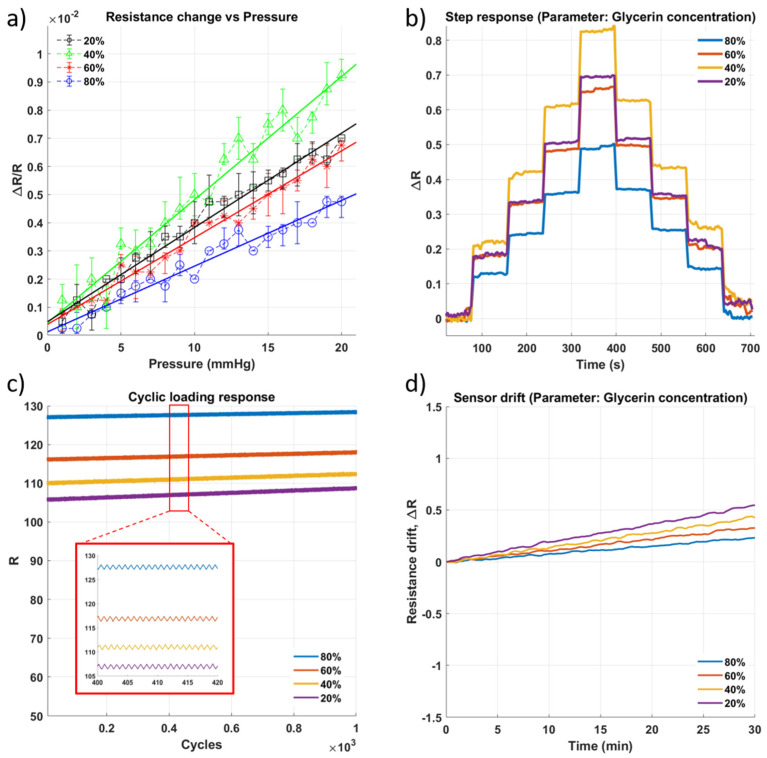
Impact of change in glycerin composition of the sensing gel on the sensor performance. (**a**) Sensitivity within the sensing range of 0–20 mmHg. (**b**) Step response of the sensor as pressure is increased in steps of approximately 5 mmHg with a 60 s hold at each pressure level. (**c**) Results of cyclic loading of the sensor for 1000 cycles of pressure at 20 mmHg (**d**) Drift of sensor for approximately 30 min.

Aloe vera gel is highly viscous, making it difficult to create a liquid-sensing medium. It also readily undergoes frothing while mixing with salt, which results in trapped air bubbles, indirectly affecting the sensing medium’s conductivity and, hence, the sensor’s performance. Hence, glycerin, a commonly used thinning agent for ionic conductive medium, was introduced. Four different concentrations were tested, from 20% to 80%, to identify the relationship between the sensor’s sensitivity and the concentration of glycerin. As the concentration of glycerin increased, the sensor sensitivity to pressure increased for up to 40% concentration (Figure 3a). Solubility of NaCl in glycerin-water mixture tends to decrease with an increase in glycerin content [29]. Since aloe vera gel is 99% water, salt readily dissolves to provide the ions for conductivity in lower glycerin concentrations. Thus, in higher glycerin concentrations, the sensing gel has lower conductivity, leading to smaller resistance changes to successive pressure input steps (Figure 3b). However, the addition of the glycerin positively impacted the sensing gel during repeated and prolonged loading, which can be seen from the cyclic loading response (Figure 3c) and drift response (Figure 3d) of the sensor. The gradient, i.e., the rate at which the resistance of the sensor changes over time for constant input, decreased as the concentration of glycerin increases. This result aligns with the observation reported in [30], that the addition of hygroscopic chemicals such as glycerin to hydrogels enhance their ambient stability and maintain conductivity for a long duration.

#### 4.2.2. Varying Geometrical Dimensions of the Sensing Channel

To identify the differences that geometry induces in the performance of the pressure sensor, a variety of geometrical designs were tested for their sensitivity, durability, and drift. Based on trials conducted by varying the concentration of glycerin in the sensing gel, a concentration of 40% was identified to provide the highest sensitivity (4.374 × 10^−4^ mmHg^−1^). Therefore, this concentration was used to fabricate the sensors tested for the impact of geometrical variation on their performance.

**Figure 4:**
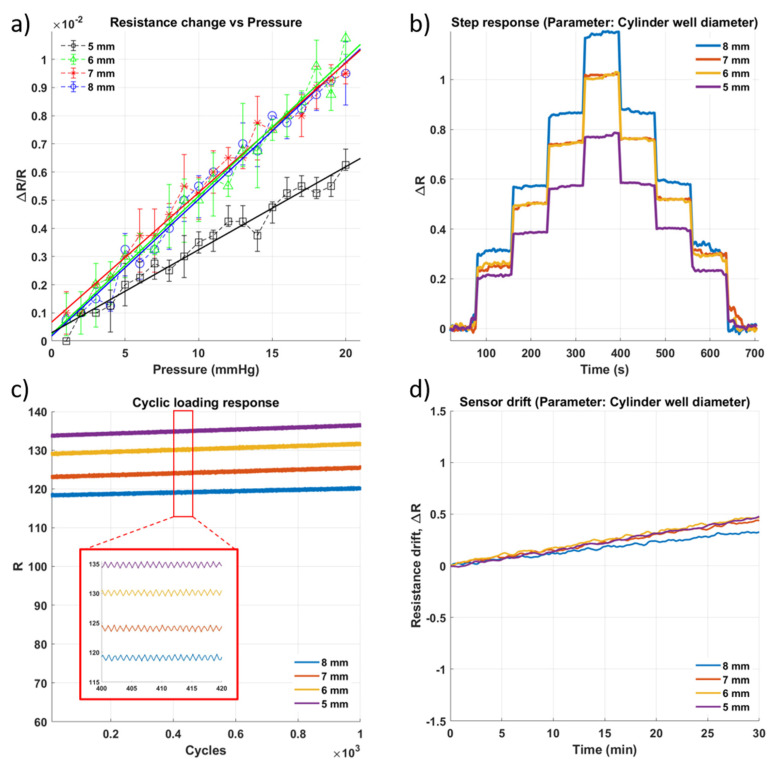
Impact of change in diameter of the cylindrical well (Figure 1(c1)) on the sensor performance. (**a**) Sensitivity within the sensing range of 0–20 mmHg. (**b**) Step response of the sensor as pressure is increased in approximately 5 mmHg with a 60 s hold at each pressure level. (**c**) Results of cyclic loading of a sensor for 1000 cycles of pressure at 20 mmHg (**d**) Drift of sensor for approximately 30 min.

The first geometric parameter is the diameter of the cylindrical central channel. Figure 4 summarizes the results from the four experiments performed on the cylindrical channel pressure sensors. The sensor with the largest diameter channel of 8 mm showed the highest sensitivity, as evidenced by the gradient of the regression line in Figure 4a. This is because a sensing channel with a larger diameter deforms to a greater extent under the same pressure compared to one with a smaller channel, which then results in larger changes in resistance of the sensor being recorded. The same can also be confirmed by referring to Figure 4b, where the response for the step increases in pressure shows that the sensor with channel diameter 8 mm has the clearest peaks as the pressure increases in 5 mmHg increments. This also indicates that the resistance change of the sensor can be discerned despite the drift shown in Figure 4d. This is essential for a good pressure sensor, as any changes in the pressure measured must always be easily identifiable. Corrections and recalibrations performed to account for the sensor drift should not adversely affect the ability of the sensor to detect pressure changes.

**Figure 5:**
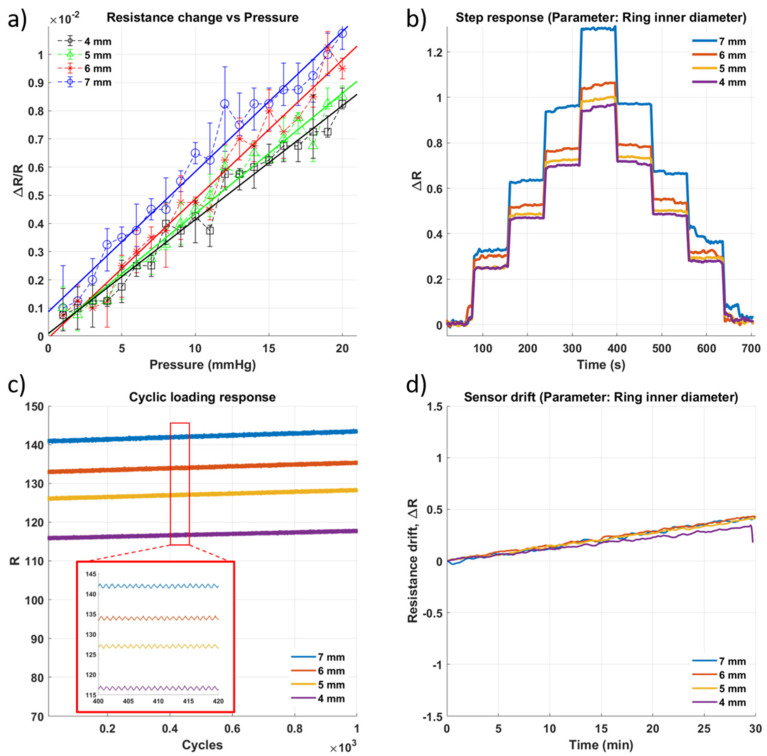
Impact of change in diameter of the inner ring of the ring-like geometry of the well (Figure 1(c2)) on the sensor performance. (**a**) Sensitivity within the sensing range of 0–20 mmHg. (**b**) Step response of the sensor as pressure is increased in steps of approximately 5 mmHg with a 60 s hold at each pressure level. (**c**) Results of cyclic loading of the sensor for 1000 cycles of pressure at 20 mmHg (**d**) Drift of sensor for approximately 30 min.

Figure 5 shows the results of testing for pressure sensors that were fabricated with ring-shaped channels with an outer diameter ($\emptyset_{o}$) of 8 mm and inner diameters ($\emptyset_{i}$) of 7, 6, 5, and 4 mm, respectively. This design was chosen as the ring shape was hypothesized to reduce the size of the channel containing the sensing gel and consequently create a disproportionately larger deformation relative to the smaller cross-sectional area of the channel. The sensitivity was highest for the sensor with the narrowest channel ($\emptyset_{i}$ = 7 mm) as it has the highest gradient in Figure 5a, as well as the most discernible levels in Figure 5b. All the sensors appear to show a comparable degradation rate during the cycling test (Figure 5c), which can be attributed to the fact that they all use the same type of sensing gel with the same concentration of glycerin. However, the ring sensors do not show any marked improvement in their sensitivity compared to the cylindrical channel sensors. One possible reason for this is that the smaller channels of the sensor make it more prone to becoming impacted by air bubbles and to the degradation of the sensing gel, which would mean that the degradation of the gel would offset any possible gain for sensitivity. Alternatively, the electrode placement combined with the ring shape could have influenced the electric field that suddenly formed within the sensing gel, which, as a result, would affect the behaviour of the sensor under pressure as well.

**Figure 6:**
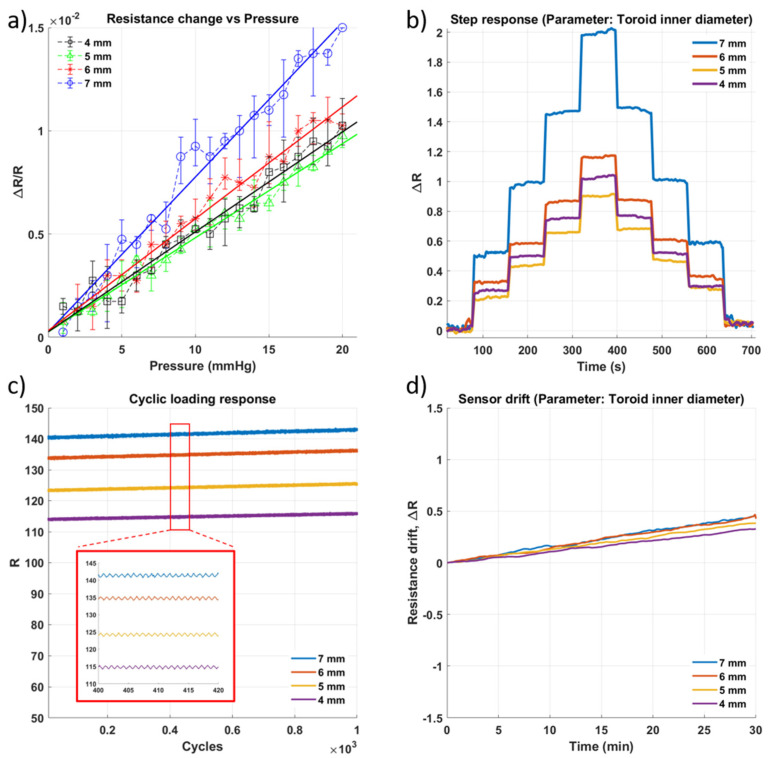
Impact of change in inner diameter of the toroid well (Figure 1(c3)) on the performance of the sensor. (**a**) Sensitivity within the sensing range of 0–20 mmHg. (**b**) Step response of the sensor as pressure is increased in steps of approximately 5 mmHg with a 60 s hold at each pressure level. (**c**) Results of cyclic loading of the sensor for 1000 cycles of pressure at 20 mmHg (**d**) Drift of sensor for approximately 30 min.

To further investigate if a reduction in the channel’s size would impact the sensor’s deformability, an alteration to the ring sensors was made. The center section of the sensor, of 3 mm in diameter, was removed from the elastomeric body of the sensor. This would allow the sensor to be more deformable as there would be less elastomer exerting elastic recoil force against the pressure that would be causing deformation of the sensor. The results of testing this type of sensor, once again with four different inner diameters, can be seen in Figure 6. This time, the sensor sensitivity with the smallest channel was much higher than that of the other sensors, even the ring-shaped sensor with the same inner diameter. This seems to indicate that the thinner the walls of the channel, the greater the sensitivity of the sensor.

**Figure 7:**
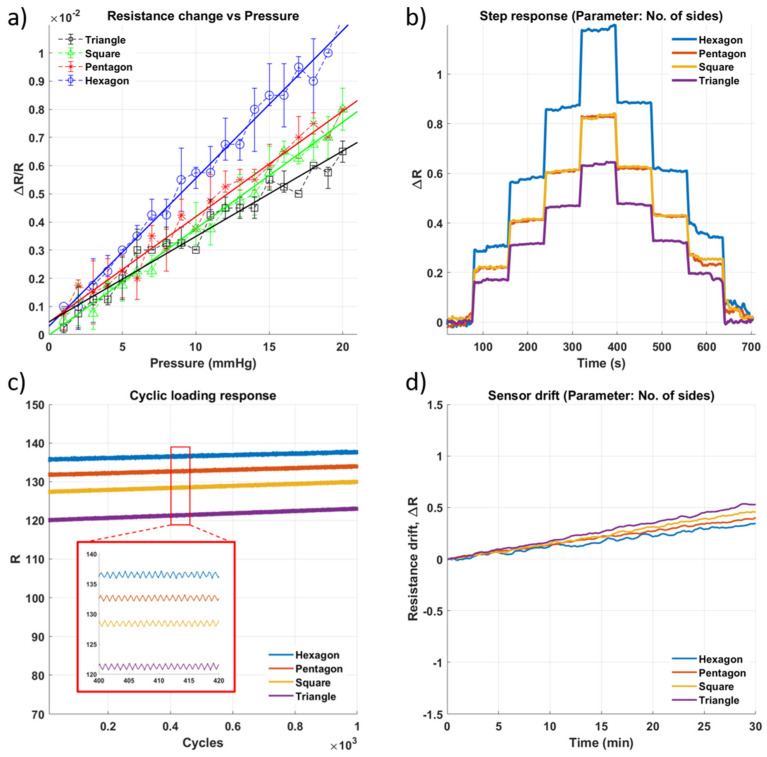
Impact of change in number of sides of the polygonal shape of the well (Figure 1(c4)) on the performance of the sensor. (**a**) Sensitivity within the sensing range of 0–20 mmHg. (**b**) Step response of the sensor as pressure is increased in steps of approximately 5 mmHg with a 60 s hold at each pressure level. (**c**) Results of cyclic loading of sensor for 1000 cycles of pressure at 20 mmHg (**d**) Drift of sensor for approximately 30 min.

Lastly, the impact of uneven deformation on the sensor’s performance was explored since the polygonal channels were expected to deform non-uniformly compared to the circular channels. The hexagonal channel, which is closest to a circular shape, had similar sensitivity to a cylindrical channel of the same size (8 mm). The triangular and square-shaped channels had low sensitivity values. One possible reason for this is that the electrodes inserted into the sensor introduced differently distributed electric fields within the sensing gel for each of the polygonal shapes, which then influenced the sensitivity of the sensor and the ease of deformation of each type of channel. These results are summarized in Figure 7.

Overall, almost all the sensor prototypes designed for parametric variation experiments provide a higher sensitivity than the AgNW sensor used in the initial experiment. This indicates that with refinements of the manufacturing process, the sensitivity of this sensor can be improved, based on the pressure-sensing application. At the same time, the sensor exhibits a drift resultant of the degradation of the ionic sensing gel. It is an unavoidable side effect of using water-based gel, since passing an electric current through the gel during its operation causes the ions in the gel to migrate to either electrode, reducing the conductivity of the solution over time. Furthermore, all the tested sensors were able to withstand a cyclic pressure of 20 mmHg for over 1000 cycles, indicating that they are physically durable. The highest sensitivity obtained was for the toroidal-shaped sensor with the thinnest channel (outer diameter = 8 mm, inner diameter = 7 mm), when using a sensing gel with a glycerin concentration of 40%. The sensitivities of all prototypes with their corresponding design parameters and values are provided in Table 1.

## 5. Conclusions

This work presents a novel aloe vera gel-based soft ionic pressure sensor for low-pressure applications. The proposed fluidic sensing medium, a mixture of glycerin, aloe vera gel and common salt (NaCl), is filled with an elastomeric substrate in a channel. The sensor exhibits a linear relationship between the pressure applied and the resistance change across its terminals, characterized by its sensitivity. We also presented sensor-design optimization for high sensitivity, based on the sensing medium composition and four different geometric parameters. A maximum sensitivity of 7.5 × 10^−4^ Ω/mmHg for the pressure range 0–20 mmHg was observed from a toroidal shaped geometry (center removed) with outer and inner diameters of 8 mm and 7 mm, respectively, filled with conductive fluid containing 40% glycerin concentration. The proposed sensor for the low-pressure range can be applied across different fields, including robotics and biomedical engineering (Ex: ICP sensing). The sensor characterization could also be modified to measure the applied force instead of pressure. In future studies, the further optimization of the channel geometry can be performed by introducing specific microstructures or multiple sensing channels to decouple multidirectional, normal or tangential forces.

## Figures and Tables

**Table 1:** Summary of sensitivity obtained from the different prototypes with their corresponding parametric values. Values in red denote the parametric values that recorded maximum sensitivity.

Design Parameter	Parametric Value and Sensitivity$\;(\times 10^{-4}\;\Omega /mmHg)$			
Glycerin concentration	80%	60%	40%	20%
	2.335	3.083	4.374	3.350
Cylinder well diameter	8 mm	7 mm	6 mm	5 mm
	4.857	4.602	4.300	2.949
Inner diameter of the ring	7 mm	6 mm	5 mm	4 mm
	4.976	4.938	4.273	4.043
Inner diameter of the Torus	7 mm	6 mm	5 mm	4 mm
	7.498	5.415	4.556	4.838
Polygon shape	Hexagon	Pentagon	Square	Triangle
	5.254	3.748	3.782	3.036

## Data Availability

The data presented in this study are available within the article.
